# Correction to ‘First Molecular Characterisation of the Lumpy Skin Disease Virus From the North African Region, Algeria and Tunisia’

**DOI:** 10.1002/vms3.70844

**Published:** 2026-03-09

Zouyed, I., Soualah, H., Djemai, S., et al. 2026. “First Molecular Characterisation of the Lumpy Skin Disease Virus From the North African Region, Algeria and Tunisia.” *Veterinary Medicine and Science* 12, no. 1: e70686. https://doi.org/10.1002/vms3.70686.

The Funding and Acknowledgments sections were accidentally omitted and should read as follows:

**Funding**

The authors acknowledge the financial support of the Ministry of Higher Education and Scientific Research through the ‘Laboratoire d’épidémiologie d'infections enzootiques des herbivores’ (LR16AGR01). This paper was also partly supported by the Federal Foreign Office Germany through the German Partnership Program on Biosecurity (Project: ‘Reducing biological proliferation risks when dealing with highly pathogenic agents in MENA countries: Middle East/ North Africa’), grant number F‐PF‐3‐OR12‐370.43#5#14/TUN.

**Acknowledgements**

The authors thank all the cattle farmers that participated in the study. They are also grateful to the support from the laboratory of parasitology at the National School of Veterinary Medicine of Sidi Thabet. Special thanks to Prof. Moez Mhadhbi and to Prof. Mohamed Aziz Darghouth for their valuable advices.

We apologize for these errors.

